# Stochastic dynamics in a delayed epidemic system with Markovian switching and media coverage

**DOI:** 10.1186/s13662-020-02894-5

**Published:** 2020-08-26

**Authors:** Chao Liu, Jane Heffernan

**Affiliations:** 1 Institute of Systems Science, Northeastern University, Wenhua Road, No. 3-11, 110004 Shenyang, China; 2 Department of Mathematics and Statistics, York University, Keele Street, No. 4700, M3J 1P3 Toronto, Canada; 3 Centre for Disease Modelling, York University, Keele Street, No. 4700, M3J 1P3 Toronto, Canada

**Keywords:** Telephone noise, Media coverage, Lévy jumps, Distributed delay, Persistence in mean, Extinction of disease

## Abstract

A stochastic SIR system with Lévy jumps and distributed delay is developed and employed to study the combined effects of Markovian switching and media coverage on stochastic epidemiological dynamics and outcomes. Stochastic Lyapunov functions are used to prove the existence of a stationary distribution to the positive solution. Sufficient conditions for persistence in mean and the extinction of an infectious disease are also shown.

## Introduction

The dynamic effects of time delays and stochastic noise on disease outcomes in populations are important research themes in mathematical epidemiology (see [1–17] and the references therein). Models incorporating systems of delay differential equations have been shown to exhibit more complex dynamics and capture more of the observed biology underlying disease transmission and persistence (see [11, 15–17] and the references therein). Studies of environmental noise in models have also been shown to capture a broader range of disease outcomes, i.e. large fluctuations in environmental noise have been shown to render a disease extinct in a model that otherwise would have shown disease progression to a unique endemic equilibrium [5, 6, 17].

Recently, a stochastic SIR epidemic system with distributed delay has been proposed [17]. Specifically, the model was used to study the effects of white noise (given by $B(t)$, representing standard Brownian motion) and a distributed delay in the infection term (incorporated using kernel $H: [0,\infty )\rightarrow [0,\infty )$, representing $L^{1}$-weak generic kernel function $H(t)=\rho e^{-\rho t}$ with $\rho >0$ such that $\int _{0}^{\infty }H(\tau )\,\mathrm{d}\tau =1$) on the extinction and persistence of a disease, given the following model structure:

1 $$ \textstyle\begin{cases} \mathrm{d}{S}(t)=[\varLambda -d_{1} S(t)-m S(t)\int ^{t}_{-\infty }H(t- \tau )I(\tau )\,\mathrm{d}\tau ]\,\mathrm{d}t+\omega S(t)\,\mathrm{d}B(t), \\ \mathrm{d}{I}(t)=[m S(t)\int ^{t}_{-\infty }H(t-\tau )I(\tau ) \,\mathrm{d}\tau -(d_{2}+\delta +c)I(t)]\,\mathrm{d}t, \\ \mathrm{d}{R}(t)=[\delta I(t)-d_{3} R(t)]\,\mathrm{d}t, \end{cases} $$

where $S(t)$, $I(t)$ and $R(t)$ represent the proportions of susceptible, infective and recovered individuals in a population, *Λ* and *δ* denote birth and recovery rates, $d_{i}$ ($i=1,2,3$) and *c* denote the natural and disease induced death rates, *m* measures the average contact rate per day, and $\omega ^{2}>0$ represents the intensity of white noise. Here, we propose an extension of this model to include Markov switching and telephone noise, Lévy jumps and media impact.

Telephone noise [18–22] (also known as telegraph noise or burst noise) can be regarded as a switching state (that is memoryless, with exponentially distributed waiting times [21, 22]) that allows for instantaneous transitions between two or more environmental regimes. By analysing observation data from the real world and performing mathematical modelling analysis, it should be noted that the birth rate of a susceptible individual is usually subject to various noises [18–20], i.e. telegraph noise. Hence, the telegraph noise is only included in the birth rate in this paper. Here, we propose some hypothesis:

1. An irreducible and continuous Markov chain $\{\beta (t),t\geq 0\}$ with finite state space $\mathbb{N}=\{1,2,\ldots ,k\}$ ($k\in Z^{+}$) is utilised to depict telephone noise. $\beta (t)$ is assumed to be generated by a transition rate matrix $(\mu _{ij})_{k\times k}$, which is
  $$ \mathbb{P}\bigl\{ \beta (\tau +\triangle \tau )=j|\beta (\tau )=i\bigr\} = \textstyle\begin{cases} \mu _{ij}\triangle \tau +o(\triangle \tau ),&i\neq j, \\ 1+\mu _{ii}\triangle \tau +o(\triangle \tau ),&i=j, \end{cases} $$
  where the transition rate from state *i* to state *j* is denoted by $\mu _{ij}\geq 0$, and $\mu _{ii}=-\sum _{i\neq j,i=1}^{k}\mu _{ij}$ holds for $i\neq j$. It follows from the irreducibility property of $\beta (t)$ that there exists a unique stationary probability distribution $\xi =(\xi _{1}, \xi _{2}, \ldots , \xi _{k})\in \mathbb{R}^{1\times k}$ subject to $\sum _{j=1}^{k}\xi _{j}=1$, $\xi _{j}>0$ hold for any $j\in \mathbb{N}$.

Recent studies have shown that Lévy jumps can effectively portray an unexpected outbreak of infectious disease and other suddenly severe perturbations arising in the real world [23–28] that cannot be accurately depicted by Brownian motion. Consequently, we consider Lévy jumps using the following hypothesis:

(H2) $S(t-)$ denotes the left limit of $S(t)$. $\mathbb{M}$ is a measurable subset of $\mathbb{R}_{+}$, *Y* denotes an independent Poisson counting measure with a Lévy measure *ψ* on $\mathbb{M}$ with $\psi (\mathbb{M})<+\infty $ satisfying $\widetilde{U}(\mathrm{d}t,\mathrm{d}u)=U(\mathrm{d}t,\mathrm{d}u)-\psi ( \mathrm{d}u)\,\mathrm{d}t$, by assuming that $\lambda (u)>-1$ and $\upsilon >0$ satisfying
  2 $$ \int _{\mathbb{M}}\bigl[\bigl(\ln \bigl(1+\lambda (u)\bigr)\bigr)\vee \ln \bigl(1+\lambda (u)\bigr)^{2}\bigr] \psi \,\mathrm{d}u\leq \upsilon .$$

Finally, it is well known that there is a profound relationship between public health issues and mass media coverage. Media reports can affect individual behaviour during an infectious disease outbreak, thus affecting the transmission of the infectious disease [29–33] and the effects of intervention strategies that are also affected by individual behaviour [34–36]. Therefore, it is necessary to consider crucial effects of media coverage on epidemiology dynamics. Based on the above analysis, some hypothesis is as follows:

(H3) A nonlinear function $m_{1}-\frac{m_{2}I(t)}{q+I(t)}$ is introduced to depict effective contact rate between susceptible and infective individual [33], $m_{1}$ represents maximal average contact rate and $\frac{m_{2}I(t)}{q+I(t)}$ denotes maximal reduced average contact rate due to public health risk warning disseminated by mass media, where $m_{1}>m_{2}>0$ and $q>0$.

Based on hypotheses (H1)–(H3), a stochastic delayed SIR system with telephone noise and media coverage is constructed as follows:

3 $$ \textstyle\begin{cases} \mathrm{d}{S}(t)= [\varLambda (\beta (t))-d_{1} S(t)- (m_{1}- \frac{m_{2}I(t)}{q+I(t)} )S(t)\int ^{t}_{-\infty }\rho e^{-\rho (t- \tau )}I(\tau )\,\mathrm{d}\tau ]\,\mathrm{d}t \\ \hphantom{\mathrm{d}{S}(t)={}}{} +\omega S(t)\,\mathrm{d}B(t)+\int _{\mathbb{M}} \lambda (u)S(t-)\widetilde{U}(\mathrm{d}t,\mathrm{d}u), \\ \mathrm{d}{I}(t)= [ (m_{1}-\frac{m_{2}I(t)}{q+I(t)} )S(t) \int ^{t}_{-\infty }\rho e^{-\rho (t-\tau )}I(\tau )\,\mathrm{d}\tau -(d_{2}+ \delta +c)I(t) ]\,\mathrm{d}t, \\ \mathrm{d}{R}(t)= [\delta I(t)-d_{3} R(t) ]\,\mathrm{d}t. \end{cases} $$

Recently, some delayed stochastic SIR systems have been used to investigate the combined dynamic effects of stochastic fluctuation and time delay on epidemiological dynamics [37–42]. Additionally, complex dynamical behaviours caused by media coverage have been investigated in stochastic epidemic systems in [29–35, 43]. To the authors’ best knowledge, combined dynamics of Markovian switching and media coverage on stochastic SIR epidemic system have not been investigated before. In the second section, stochastically ultimate boundedness of the solution is studied. Existence and uniqueness of globally positive solution to the proposed system are investigated. Existence of a stationary distribution to the positive solution is discussed. In the third section, sufficient conditions for persistence in mean of each individual and extinction of infectious disease are discussed. Numerical simulations are supported to illustrate the main theoretical results. Finally, this paper ends with a conclusion.

## Qualitative analysis of stationary distribution

Setting $W(t)=\int ^{t}_{-\infty }\rho e^{-\rho (t-\tau )}I(\tau )\,\mathrm{d} \tau $, it follows from the linear chain technique [44] that system (3) can be written as

4 $$ \textstyle\begin{cases} \mathrm{d}{S}(t)= [\varLambda (\beta (t))-d_{1} S(t)- (m_{1}- \frac{m_{2}I(t)}{q+I(t)} )S(t)W(t) ]\,\mathrm{d}t \\ \hphantom{\mathrm{d}{S}(t)= {}}{} +\omega S(t)\,\mathrm{d}B(t)+\int _{\mathbb{M}} \lambda (u)S(t-)\widetilde{U}(\mathrm{d}t,\mathrm{d}u), \\ \mathrm{d}{I}(t)= [ (m_{1}-\frac{m_{2}I(t)}{q+I(t)} )S(t)W(t)-(d_{2}+ \delta +c)I(t) ]\,\mathrm{d}t, \\ \mathrm{d}{R}(t)= [\delta I(t)-d_{3} R(t) ]\,\mathrm{d}t, \\ \mathrm{d}W(t)=\rho [I(t)-W(t) ]\,\mathrm{d}t. \end{cases} $$

For every finite state space $k\in \mathbb{N}$ defined in (H1), it follows from the Markov chain law that system (4) can be investigated as a hybrid system switching among the following subsystems:

5 $$ \textstyle\begin{cases} \mathrm{d}{S}(t)= [\varLambda (k)-d_{1} S(t)- (m_{1}- \frac{m_{2}I(t)}{q+I(t)} )S(t)W(t) ]\,\mathrm{d}t \\ \hphantom{\mathrm{d}{S}(t)={}}{} +\omega S(t)\,\mathrm{d}B(t)+\int _{\mathbb{M}} \lambda (u)S(t-)\widetilde{U}(\mathrm{d}t,\mathrm{d}u), \\ \mathrm{d}{I}(t)= [ (m_{1}-\frac{m_{2}I(t)}{q+I(t)} )S(t)W(t)-(d_{2}+ \delta +c)I(t) ]\,\mathrm{d}t, \\ \mathrm{d}{R}(t)= [\delta I(t)-d_{3} R(t) ]\,\mathrm{d}t, \\ \mathrm{d}W(t)=\rho [I(t)-W(t) ]\,\mathrm{d}t.\end{cases} $$

First, we discuss stochastically ultimate boundedness of the solution. Existence and uniqueness of globally positive solutions to the proposed system are also studied.

### Lemma 2.1

*If hypotheses* (H2), (H3) *hold and* $(m_{1},m_{2})\in \mathcal{D}_{1}$, $\mathcal{D}_{1}$*is defined in* (6) *and* $Q_{1}(\gamma )$*is defined in* (49), *then system* (5) *with any initial value* $(S(0), I(0), R(0), W(0), k)\in \mathbb{R}_{+}^{4}\times \mathbb{N}$*is stochastically ultimately bounded*.

6 $$ \mathcal{D}_{1}=\left \{ (m_{1},m_{2})\Big|0< m_{2}< m_{1}< \frac{d_{2}+\delta +c}{Q_{1}(\gamma )}\right . \biggr\} .$$

### Proof

The proof of Lemma 2.1 can be found in Appendix A. □

### Lemma 2.2

*If hypotheses* (H2), (H3) *hold and* $(m_{1},m_{2})\in \mathcal{D}_{1}$, *then for any initial value* $(S(0), I(0), R(0), W(0), k)\in \mathbb{R}_{+}^{4}\times \mathbb{N}$, *system* (5) *has a unique global positive solution for all* $t\geq 0$*almost surely*.

### Proof

The proof of Lemma 2.2 can be found in Appendix B. □

In the following part, we will consider the existence of a stationary distribution to the positive solution (which is a stationary Markov process) by constructing appropriately Lyapunov functions.

### Theorem 2.3

*If hypotheses* (H2) *and* (H3) *hold*, $(m_{1},m_{2})\in \mathcal{D}_{1}\cap \mathcal{D}_{2}$, *where* $\mathcal{D}_{1}$*and* $\mathcal{D}_{2}$*are defined in* (6) *and* (7), *then* $(S(t), I(t), R(t), W(t))$*of system* (5) *is a stationary Markov process*.

7 $$ \mathcal{D}_{2}=\left \{ (m_{1},m_{2})\left| \begin{aligned} &m_{1}q-m_{2}Q(\varepsilon )>0, \\ &\frac{2m_{1}\varLambda (k)}{d_{2}+\delta +c}+ \biggl(2m_{2}+ \frac{m_{1}}{d_{1}} \biggr)Q(\varepsilon )+ \frac{qm_{2}\varLambda (k)Q(\varepsilon )}{A_{1}} \\ &\quad < 2d_{1}+\frac{\varLambda (k)}{d_{1}A_{1}Q(\varepsilon )} \biggl[d_{1} \biggl(d_{3}+\delta +\frac{\omega ^{2}}{2}-c-d_{2} \biggr)+A_{1} \biggr]-1\end{aligned} \right . \right \}, $$

*where* $A_{1}=\sqrt{ (m_{1}-\frac{m_{2}Q(\varepsilon )}{q} ) (\delta +\frac{\omega ^{2}}{2} )}$.

### Proof

If $m_{1}q-m_{2}Q(\varepsilon )>0$, then we define

$$\begin{aligned} Z_{1}(S, I, R, W) =&\frac{\rho A_{1}Q(\varepsilon )}{\varLambda (k)} \biggl(2\ln S- \frac{S}{d_{1}Q(\varepsilon )} \biggr)-\rho (\ln I+\ln R) \\ &{}- \biggl(\delta +\frac{\omega ^{2}}{2} \biggr)\ln W+ \frac{2A_{1}Q(\varepsilon ) [\rho (I+R)+W]}{\delta \varLambda (k)}. \end{aligned}$$

According to the biological interpretations of the second equation and fourth equation in system (5), it can be obtained that

8 $$ \textstyle\begin{cases} \frac{2\varLambda (k)}{S}< \frac{2\varLambda (k)W}{d_{2}+\delta +c} ( \frac{m_{1}}{I}-\frac{m_{2}}{q+I} )< \frac{2m_{1}\varLambda (k)W}{(d_{2}+\delta +c)I}, \\ \frac{2m_{1}\varLambda (k)W}{(d_{2}+\delta +c)I}< \frac{2m_{1}\varLambda (k)}{d_{2}+\delta +c}. \end{cases} $$

Using Itô’s formula [45] on $Z_{1}(S, I, R, W)$, it follows from (8) and Lemma 2.1 that

9 $$\begin{aligned} \mathcal{L}Z_{1} =&\frac{\rho A_{1}Q(\varepsilon )}{\varLambda (k)} \biggl(\frac{2}{S}-\frac{1}{d_{1}Q(\varepsilon )} \biggr) \biggl[ \varLambda (k)-d_{1}S- \biggl(m_{1}-\frac{m_{2}I}{q+I} \biggr)SW \biggr] \\ &{}+\frac{2\rho A_{1}Q(\varepsilon )}{\delta \varLambda (k)} \biggl[ \biggl(m_{1}- \frac{m_{2}I}{q+I} \biggr)SW-(d_{2}+c-1)I-d_{3}R-W \biggr] \\ &{}-\rho \biggl[ \biggl(\frac{m_{1}}{I}-\frac{m_{2}}{q+I} \biggr)SW-d_{2}+d_{3}+c+ \delta \biggr] \\ &{}-\rho \biggl[\frac{\delta I}{R}+ \biggl(\delta + \frac{\omega ^{2}}{2} \biggr) \biggl(\frac{I}{W}-1 \biggr) \biggr] \\ \leq &\frac{\rho A_{1}Q(\varepsilon )}{\varLambda (k)} \biggl[ \frac{2m_{1}\varLambda (k)}{d_{2}+\delta +c}+1+ \biggl(2m_{2}+ \frac{m_{1}}{d_{1}} \biggr)Q(\varepsilon )-2d_{1}- \frac{\varLambda (k)}{d_{1}Q(\varepsilon )} \biggr] \\ &{}+\rho \biggl[\frac{m_{2}Q^{2}(\varepsilon )}{q}+d_{2}+c-d_{3}- \delta -\frac{\omega ^{2}}{2} \biggr]+ \frac{\rho A_{1}Q(\varepsilon )}{\varLambda (k)}\frac{2m_{1}IW}{\delta } \\ :=&-{A}_{2}+\frac{\rho A_{1}Q(\varepsilon )}{\varLambda (k)} \frac{2m_{1}IW}{\delta }. \end{aligned}$$

Define $Z_{2}(S, I, R, W)=\frac{1}{\eta +2} (S+I+R+\frac{W}{\rho } )^{\eta +2}$, where *η* is sufficiently small and chosen randomly from $\eta \in (0, \frac{d_{2}+\delta +c-\frac{\omega ^{2}}{2}-\int _{\mathbb{M}}[\lambda (u) -\ln (1+\lambda (u))]\psi \,\mathrm{d}u}{d_{2}+\delta +c+\frac{\omega ^{2}}{2} +\int _{\mathbb{M}}[\lambda (u)-\ln (1+\lambda (u))]\psi \,\mathrm{d}u} )$.

By using Itô’s formula [45] on $Z_{2}(S, I, R, W)$, it follows from simple computations and Lemma 2.1 that

10 $$\begin{aligned} \mathcal{L}Z_{2} =& \biggl(S+I+R+ \frac{W}{\rho } \biggr)^{\eta +1} \bigl[\varLambda (k)-d_{1}S-(d_{2}+c-1)I-d_{3}R-W \bigr] \\ &{}+\frac{\eta +1}{2} \biggl(S+I+R+\frac{W}{\rho } \biggr)^{\eta } \biggl[ \omega ^{2}S^{2}+ \int _{\mathbb{M}}S(u)\bigl[\lambda (u)-\ln \bigl(1+\lambda (u)\bigr) \bigr] \psi \,\mathrm{d}u \biggr] \\ \leq &\varLambda (k) \biggl(S+I+R+\frac{W}{\rho } \biggr)^{\eta +1}-d_{1}S^{ \eta +2}-(d_{2}+c-1)I^{\eta +2}-d_{3}R^{\eta +2}- \frac{W^{\eta +2}}{\rho ^{\eta +1}} \\ &{}+\frac{\eta +1}{2} \biggl(S+I+R+\frac{W}{\rho } \biggr)^{\eta } \biggl[ \omega ^{2}S^{2}+ \int _{\mathbb{M}}S(u)\bigl[\lambda (u)-\ln \bigl(1+\lambda (u)\bigr) \bigr] \psi \,\mathrm{d}u \biggr] \\ \leq &-\frac{d_{1}S^{\eta +2}}{2}-(d_{2}+c-1)\eta I^{\eta +2}-d_{3} \eta R^{\eta +2}-\frac{W^{\eta +2}}{2\rho ^{\eta +1}}+A_{3} \\ &{}+\frac{\eta +1}{2} \biggl(S+I+R+\frac{W}{\rho } \biggr)^{\eta } \bigl( \omega ^{2}S^{2}+\upsilon \bigr), \end{aligned}$$

where

$$\begin{aligned} A_{3} =&\sup_{(S,I,R,W)\in \mathbb{R}_{+}^{4}} \biggl\{ - \frac{d_{1}S^{\eta +2}}{2}-(d_{2}+c-1) (1-\eta )I^{\eta +2}-d_{3}(1- \eta )R^{\eta +2}-\frac{W^{\eta +2}}{2\rho ^{\eta +1}} \\ &{}+\varLambda (k) \biggl(S+I+R+\frac{W}{\rho } \biggr)^{\eta +1}+ \frac{\eta +1}{2} \biggl(S+I+R+\frac{W}{\rho } \biggr)^{\eta } \bigl( \omega ^{2}S^{2}+\upsilon \bigr) \biggr\} . \end{aligned}$$

Following the above analysis, we define functions $f_{j}(S,I,R,W)$ ($j=1,2,3$) and $Z_{31}(S,I, R,W)$ as follows:

$$\begin{aligned}& f_{1}(S)=-\frac{d_{1}S^{\eta +2}}{2},\qquad f_{2}(I,R)=-(d_{2}+c-1) \eta I^{\eta +2}-d_{3}\eta R^{\eta +2}, \\& f_{3}(S,I,R,W)=-\frac{W^{\eta +2}}{2\rho ^{\eta +1}}+A_{3}+\rho , \\& Z_{31}(S,I,R,W)=-\varphi Z_{1}(S,I,R,W)+Z_{2}(S,I,R,W)- \ln W, \end{aligned}$$

where the constant $\varphi >0$ satisfies $-\varphi A_{2}+\sum _{j=1}^{3}\sup_{t\geq 0}f_{j}(S,I,R,W)\leq -2$ and $A_{2}$ has been defined in (9).

Note that $Z_{31}(S,I,R,W)$ is a continuous function and tends to the boundary of $\mathbb{R}_{+}^{4}$ infinity when $\|(S,I,R,W)\|\rightarrow \infty $. Consequently, it is easy to show that there exists an extreme point $(\tilde{S}, \tilde{I}, \tilde{R}, \tilde{W})$ for $Z_{31}$ in the interior of $\mathbb{R}_{+}^{4}$.

By defining a nonnegative function

$$ Z_{3}(S,I,R,W)=\varphi Z_{1}(S,I,R,W)+Z_{2}(S,I,R,W)- \ln W-Z_{31}( \tilde{S}, \tilde{I}, \tilde{R}, \tilde{W}). $$

Based on (9) and (10), it can be obtained that

$$\begin{aligned} \mathcal{L}Z_{3} \leq &-\varphi A_{2}+ \frac{\varphi \rho A_{1}Q(\varepsilon )}{\varLambda (k)} \frac{2m_{1}IW}{\delta }-\frac{d_{1}S^{\eta +2}}{2}-(d_{2}+c-1) \eta I^{ \eta +2}-d_{3}\eta R^{\eta +2} \\ &{}-\frac{W^{\eta +2}}{2\rho ^{\eta +1}}+\frac{\eta +1}{2} \biggl(S+I+R+ \frac{W}{\rho } \biggr)^{\eta } \bigl(\omega ^{2}S^{2}+ \upsilon \bigr)+A_{3}- \frac{\rho I}{W}+\rho \\ =&\sum _{j=1}^{3}f_{j}(S,I,R,W)-\varphi A_{2}+ \frac{\varphi \rho A_{1}Q(\varepsilon )}{\varLambda (k)} \frac{2m_{1}IW}{\delta }+A_{3}- \frac{\rho I}{W}+\rho . \end{aligned}$$

Additionally, it can be shown that if $(m_{1},m_{2})\in \mathcal{D}_{1}\cap \mathcal{D}_{2}$, then

11 $$ \mathcal{L}Z_{3}\leq \sum _{j=1}^{3}\sup_{t\geq 0} f_{j}(S,I,R,W)- \varphi A_{2}\leq -2 $$

holds for either $S\rightarrow 0^{+}$ or $I\rightarrow 0^{+}$ or $R\rightarrow 0^{+}$. Furthermore,

12 $$ \textstyle\begin{cases} \mathcal{L}Z_{3}\leq \mathcal{L}Z_{3}(S,I,R,0)\rightarrow -\infty ,& W\rightarrow 0^{+}, \\ \mathcal{L}Z_{3}\leq \mathcal{L}Z_{3}(+\infty ,I,R,W)\rightarrow - \infty , & S\rightarrow +\infty , \\ \mathcal{L}Z_{3}\leq \mathcal{L}Z_{3}(S,+\infty ,R,W)\rightarrow - \infty , & I\rightarrow +\infty , \\ \mathcal{L}Z_{3}\leq \mathcal{L}Z_{3}(S,I,+\infty ,W)\rightarrow - \infty , & R\rightarrow +\infty , \\ \mathcal{L}Z_{3}\leq \mathcal{L}Z_{3}(S,I,R,+\infty )\rightarrow - \infty , & W\rightarrow +\infty . \end{cases} $$

It follows from (11), (12) and simple computations that there exists a sufficiently small positive constant $\epsilon >0$ such that $\mathcal{L}Z_{3}(S,I,R,W)\leq -1$ holds for any $(S,I,R,W)\in \mathbb{R}_{+}^{4}\setminus \varOmega $, where $\varOmega = (\epsilon ,\frac{1}{\epsilon } )\times ( \epsilon ,\frac{1}{\epsilon } )\times (\epsilon , \frac{1}{\epsilon } )\times (\epsilon ,\frac{1}{\epsilon } )$.

Based on Lemma 2.1 [10], it is straightforward to show that there exists a solution of system (5), which is a stationary Markov process. □

## Permanence in mean and extinction of disease

For the deterministic version of system (5), i.e., system (5) without Brownian motion and Lévy jumps, the endemic equilibrium $(S^{*}, I^{*}, R^{*}, W^{*})$ is as follows:

$$ S^{*}=\frac{(d_{2}+\delta +c)(q+I^{*})}{m_{1}q+(m_{1}-m_{2})I^{*}},\qquad R^{*}= \frac{\delta I^{*}}{d_{3}},\qquad W^{*}=I^{*}, $$

where $I^{*}$ satisfies

13 $$\begin{aligned}& (m_{1}-m_{2}) (d_{2}+\delta +c)I^{2}+\bigl[(d_{2}+\delta +c) (d_{1}+m_{1}q)-(m_{1}-m_{2}) \varLambda (k)\bigr]I \\& \quad {}+q\bigl[d_{1}(d_{2}+\delta +c)-m_{1} \varLambda (k)\bigr]=0. \end{aligned}$$

Based on the formulation of endemic equilibrium, it follows from the Vieta theorem that there exists a unique endemic equilibrium provided $(m_{1},m_{2})\in \mathcal{D}_{3}$, and $\mathcal{D}_{3}$ is defined as follows:

14 $$ \mathcal{D}_{3}=\biggl\{ (m_{1},m_{2})\Big| \begin{aligned} m_{1}>\max \biggl\{ m_{2}, \frac{d_{1}(d_{2}+\delta +c)}{\varLambda (k)}, \frac{m_{2}\varLambda (k)+d_{1}(d_{2}+\delta +c)}{\varLambda (k)-q(d_{2}+\delta +c)} \biggr\} \end{aligned} \biggr\} . $$

In the following, we discuss permanence in mean of each individual and disease extinction in system (5). Some corresponding practical interpretations can be found in [17] and the references therein.

### Theorem 3.1

*For any initial value* $(S(0), I(0), R(0), W(0), k)\in \mathbb{R}_{+}^{4}\times \mathbb{N}$, *if hypotheses* (H2) *and* (H3) *hold*, $(m_{1},m_{2})\in \mathcal{D}_{1}\cap \mathcal{D}_{2}\cap \mathcal{D}_{3} \cap \mathcal{D}_{4}$, $S^{*2}C_{6}>C_{5}$, $I^{*2}C_{6}>C_{5}$, $R^{*2}C_{6}>C_{5}$, $W^{*2}C_{6}>C_{5}$, *then system* (5) *is permanent in mean*, *where* $\mathcal{D}_{4}$*is defined in* (15), *and* $C_{5}$*and* $C_{6}$*are defined in* (20) *and* (21).

15 $$ D_{4}=\left \{ (m_{1},m_{2})|m_{1} \bigl(q+Q(\varepsilon )\bigr) \bigl(q+I^{*}\bigr)< m_{2}I^{*} \bigl(1+P( \varepsilon )\bigr) \right . \bigr\} .$$

### Proof

Firstly, we construct $U_{1}(t)=\frac{(S(t)-S^{*})^{2}}{2}$. Using Itô’s formula to system (5), we obtain

$$\begin{aligned} \mathrm{d}U_{1}(t) =&\bigl(S(t)-S^{*}\bigr) \biggl[d_{1}\bigl(S^{*}-S(t)\bigr)+ \biggl(m_{1}- \frac{m_{2}I^{*}}{q+I^{*}} \biggr) \bigl(S^{*}W^{*}-S(t)W(t) \bigr) \biggr] \,\mathrm{d}t \\ &{}+\bigl(S(t)-S^{*}\bigr) \biggl[\frac{\omega ^{2}}{2}+ \int _{\mathbb{M}}\bigl[ \lambda (u)-\ln \bigl(1+\lambda (u)\bigr) \bigr]\psi \,\mathrm{d}u \biggr]\,\mathrm{d}t+\bigl(S(t)-S^{*}\bigr) \omega \,\mathrm{d}B(t) \\ &{}+\bigl(S(t)-S^{*}\bigr) \int _{\mathbb{M}}\bigl[\lambda (u)S(t-)-\ln \bigl(1+\lambda (u) \bigr)\bigr] \widetilde{U}(\mathrm{d}t,\mathrm{d}u) \\ \leq &- \biggl(d_{1}+m_{1}W^{*}- \frac{m_{2}I^{*}W^{*}}{q+I^{*}} \biggr) \bigl(S(t)-S^{*}\bigr)^{2} \,\mathrm{d}t \\ &{}+\frac{m_{2}qS(t)W(t)}{(q+I^{*})(q+I(t))}\bigl(S(t)-S^{*}\bigr) \bigl(I(t)-I^{*}\bigr) \,\mathrm{d}t \\ &{}+\frac{S(t)(S(t)W(t)+S^{*}W^{*})[q+(m_{2}+1)I^{*}]}{q+I^{*}} \,\mathrm{d}t \\ &{}+\bigl(S(t)-S^{*}\bigr) \biggl[\frac{\omega ^{2}}{2}+ \int _{\mathbb{M}}\bigl[ \lambda (u)-\ln \bigl(1+\lambda (u)\bigr) \bigr]\psi \,\mathrm{d}u \biggr]\,\mathrm{d}t \\ &{}+\bigl(S(t)-S^{*}\bigr)\omega \,\mathrm{d}B(t)+ \bigl(S(t)-S^{*}\bigr) \int _{\mathbb{M}}\bigl[ \lambda (u)S(t-)-\ln \bigl(1+\lambda (u) \bigr)\bigr]\widetilde{U}(\mathrm{d}t, \mathrm{d}u) \\ =&\mathcal{L}U_{1}\,\mathrm{d}t+\bigl(S(t)-S^{*}\bigr) \biggl[ \frac{\omega ^{2}}{2}+ \int _{\mathbb{M}}\bigl[\lambda (u)-\ln \bigl(1+\lambda (u)\bigr) \bigr] \psi \,\mathrm{d}u \biggr]\,\mathrm{d}t \\ &{}+\bigl(S(t)-S^{*}\bigr)\omega \,\mathrm{d}B(t)+ \bigl(S(t)-S^{*}\bigr) \int _{\mathbb{M}}\bigl[ \lambda (u)S(t-)-\ln \bigl(1+\lambda (u) \bigr)\bigr]\widetilde{U}(\mathrm{d}t, \mathrm{d}u). \end{aligned}$$

When hypotheses (H2) and (H3) hold, it follows from Lemma 2.1 that

16 $$\begin{aligned} \mathcal{L}U_{1} \leq &- \biggl(d_{1}+m_{1}W^{*}- \frac{m_{2}I^{*}W^{*}}{q+I^{*}} \biggr) \bigl(S(t)-S^{*}\bigr)^{2} \\ &{}+\frac{m_{2}qQ^{2}(\varepsilon )}{(q+I^{*})(q+P(\varepsilon ))}\bigl(S(t)-S^{*}\bigr) \bigl(I(t)-I^{*}\bigr) \\ &{}+ \frac{Q(\varepsilon )(Q^{2}(\varepsilon )+S^{*}W^{*})[q+(m_{2}+1)I^{*}]}{q+I^{*}}+S^{*} \biggl(\frac{\omega ^{2}}{2}+ \upsilon \biggr), \end{aligned}$$

where $Q(\varepsilon )$ and $P(\varepsilon )$ have been defined in Lemma 2.1.

Construct the function $U_{2}(t)=I(t)-I^{*}-I^{*}\ln \frac{I(t)}{I^{*}}$. By using Itô’s formula to system (5), it follows from Lemma 2.1 that

17 $$\begin{aligned} \mathrm{d}U_{2}(t) =& \bigl(I(t)-I^{*}\bigr) \biggl[m_{1} \biggl( \frac{S(t)W(t)}{I(t)}-\frac{S^{*}W^{*}}{I^{*}} \biggr)-m_{2} \biggl( \frac{S(t)W(t)}{q+I(t)}-\frac{S^{*}W^{*}}{q+I^{*}} \biggr) \biggr] \,\mathrm{d}t \\ =&- \biggl[\frac{m_{1}S(t)W(t)}{I^{*}I(t)}- \frac{m_{2}S(t)W(t)}{(q+I(t)(q+I^{*}))} \biggr] \bigl(I(t)-I^{*}\bigr)^{2} \,\mathrm{d}t \\ &{}+W^{*} \biggl[\frac{m_{1}}{I^{*}}- \frac{m_{2}(1+I(t))}{(q+I(t))(q+I^{*})} \biggr]\bigl(S(t)-S^{*}\bigr) \bigl(I(t)-I^{*}\bigr) \, \mathrm{d}t \\ &{}+S(t) \biggl[\frac{m_{1}}{I^{*}}- \frac{m_{2}(1+I(t))}{(q+I(t))(q+I^{*})} \biggr] \bigl(I(t)-I^{*}\bigr) \bigl(W(t)-W^{*}\bigr) \, \mathrm{d}t \\ =&- \frac{qm_{1}(q+I(t)+I^{*})+(m_{1}-m_{2})I^{*}I(t)}{(q+I(t))(q+I^{*})I^{*}I(t)}S(t)W(t) \bigl(I(t)-I^{*} \bigr)^{2} \,\mathrm{d}t \\ &{}+W^{*} \biggl[\frac{m_{1}}{I^{*}}- \frac{m_{2}(1+I(t))}{(q+I(t))(q+I^{*})} \biggr]\bigl(S(t)-S^{*}\bigr) \bigl(I(t)-I^{*}\bigr) \, \mathrm{d}t \\ &{}+S(t) \biggl[\frac{m_{1}}{I^{*}}- \frac{m_{2}(1+I(t))}{(q+I(t))(q+I^{*})} \biggr] \bigl(I(t)-I^{*}\bigr) \bigl(W(t)-W^{*}\bigr) \, \mathrm{d}t \\ \leq &- \frac{qm_{1}(q+I^{*}+P(\varepsilon ))+(m_{1}-m_{2})P(\varepsilon )I^{*}}{(q+Q(\varepsilon ))(q+I^{*})Q(\varepsilon )I^{*}}P^{2}( \varepsilon ) \bigl(I(t)-I^{*}\bigr)^{2}\,\mathrm{d}t \\ &{}+W^{*} \biggl[\frac{m_{1}}{I^{*}}- \frac{m_{2}(1+P(\varepsilon ))}{(q+Q(\varepsilon ))(q+I^{*})} \biggr]\bigl(S(t)-S^{*}\bigr) \bigl(I(t)-I^{*}\bigr) \, \mathrm{d}t \\ &{}+Q(\varepsilon ) \biggl[\frac{m_{1}}{I^{*}}- \frac{m_{2}(1+P(\varepsilon ))}{(q+Q(\varepsilon ))(q+I^{*})} \biggr] \bigl(I(t)-I^{*}\bigr) \bigl(W(t)-W^{*}\bigr) \, \mathrm{d}t, \end{aligned}$$

where $Q(\varepsilon )$ and $P(\varepsilon )$ have been defined in Lemma 2.1.

According to similar arguments mentioned above, we will establish two functions $U_{3}(t)=\frac{(R(t)-R^{*})^{2}}{2}$, $U_{4}(t)=\frac{(W(t)-W^{*})^{2}}{2}$, and using Itô’s formula to system (5), it can be obtained that

18 $$ \textstyle\begin{cases} \mathrm{d}U_{3}(t)\leq - \frac{\delta I^{*}(R(t)-R^{*})^{2}}{R^{*}Q(\varepsilon )}\,\mathrm{d}t+ \frac{\delta (Q^{2}(\varepsilon )+R^{*}I^{*})}{P(\varepsilon )} \,\mathrm{d}t, \\ \mathrm{d}U_{4}(t)\leq - \frac{\rho I^{*}(W(t)-W^{*})^{2}}{W^{*}Q(\varepsilon )}\,\mathrm{d}t+ \frac{\rho (W(t)-W^{*})(I(t)-I^{*})}{P(\varepsilon )}\,\mathrm{d}t, \end{cases} $$

where $Q(\varepsilon )$ and $P(\varepsilon )$ have been defined in Lemma 2.1. Now, we define

$$ U_{5}(t)= \frac{W^{*}(q+I^{*})(q+P(\varepsilon ))C_{0}}{m_{2}qQ^{2}(\varepsilon )}U_{1}(t)+U_{2}(t)+U_{3}(t)+ \frac{P(\varepsilon )Q(\varepsilon )C_{0}}{\rho }U_{4}(t), $$

where $C_{0}=\frac{m_{2}(1+P(\varepsilon ))}{(q+Q(\varepsilon ))(q+I^{*})}- \frac{m_{1}}{I^{*}}$.

It follows from (16), (17), (18) and simple computations that

19 $$\begin{aligned} \mathcal{L}U_{5}(t) \leq &- \frac{W^{*}(q+I^{*})(q+P(\varepsilon ))C_{0}}{m_{2}qQ^{2}(\varepsilon )} \biggl(d_{1}+m_{1}W^{*}- \frac{m_{2}I^{*}W^{*}}{q+I^{*}} \biggr) \bigl(S(t)-S^{*}\bigr)^{2} \\ &{}- \frac{P^{2}(\varepsilon )[qm_{1}(q+I^{*}+P(\varepsilon ))+(m_{1}-m_{2})P(\varepsilon )I^{*}]}{(q+Q(\varepsilon ))(q+I^{*})Q(\varepsilon )I^{*}}\bigl(I(t)-I^{*}\bigr)^{2} \\ &{}-\frac{\delta I^{*}}{R^{*}Q(\varepsilon )}\bigl(R(t)-R^{*}\bigr)^{2}- \frac{I^{*}P(\varepsilon )C_{0}}{W^{*}}\bigl(W(t)-W^{*}\bigr)^{2} \\ &{}+ \frac{Q(\varepsilon )(Q^{2}(\varepsilon )+S^{*}W^{*})[q+(m_{2}+1)I^{*}]}{q+I^{*}} \\ &{}+\frac{\delta (Q^{2}(\varepsilon )+R^{*}I^{*})}{P(\varepsilon )}+S^{*} \biggl(\frac{\omega ^{2}}{2}+ \upsilon \biggr). \end{aligned}$$

By integrating both sides of (19) from 0 to *t* and performing expectations, it yields

20 $$\begin{aligned} \mathbb{E}U_{5}(t)- \mathbb{E}U_{5}(0) \leq &-C_{1}\mathbb{E} \int _{0}^{t}\bigl[S( \tau )-S^{*} \bigr]^{2}\,\mathrm{d}\tau -C_{2}\mathbb{E} \int _{0}^{t}\bigl[I(\tau )-I^{*} \bigr]^{2} \,\mathrm{d}\tau \\ &{}-C_{3}\mathbb{E} \int _{0}^{t}\bigl[R(\tau )-R^{*} \bigr]^{2}\,\mathrm{d}\tau -C_{4} \mathbb{E} \int _{0}^{t}\bigl[W(\tau )-W^{*} \bigr]^{2}\,\mathrm{d}\tau +C_{5}t, \end{aligned}$$

where $C_{j}$ ($j=1,2,3,4,5$) are defined as follows:

$$\begin{aligned}& C_{1}= \frac{W^{*}(q+I^{*})(q+P(\varepsilon ))C_{0}}{m_{2}qQ^{2}(\varepsilon )} \biggl(d_{1}+m_{1}W^{*}-\frac{m_{2}I^{*}W^{*}}{q+I^{*}} \biggr), \\& C_{2}= \frac{P^{2}(\varepsilon )[qm_{1}(q+I^{*}+P(\varepsilon ))+(m_{1}-m_{2})P(\varepsilon )I^{*}]}{(q+Q(\varepsilon ))(q+I^{*})Q(\varepsilon )I^{*}}, \\& C_{3}=\frac{\delta I^{*}}{R^{*}Q(\varepsilon )}, \\& C_{4}=\frac{I^{*}P(\varepsilon )C_{0}}{W^{*}}, \\& C_{5}= \frac{\delta (Q^{2}(\varepsilon )+R^{*}I^{*})}{P(\varepsilon )}+ \frac{Q(\varepsilon )(Q^{2}(\varepsilon )+S^{*}W^{*})[q+(m_{2}+1)I^{*}]}{q+I^{*}}+S^{*} \biggl(\frac{\omega ^{2}}{2}+\upsilon \biggr). \end{aligned}$$

If $(m_{1},m_{2})\in \mathcal{D}_{1}\cap \mathcal{D}_{2}\cap \mathcal{D}_{3} \cap \mathcal{D}_{4}$, then

21 $$ \limsup_{t\rightarrow \infty }\frac{1}{t} \mathbb{E} \int _{0}^{t}\bigl[\bigl(S( \tau )-S^{*}\bigr)^{2}+\bigl(I(\tau )-I^{*} \bigr)^{2}+\bigl(R(\tau )-R^{*}\bigr)^{2}+ \bigl(W(\tau )-W^{*}\bigr)^{2}\bigr] \,\mathrm{d}\tau \leq \frac{C_{5}}{C_{6}},$$

where $C_{6}=\min_{j=1,2,3,4}\{C_{j}\}$. Further computations yield that

$$ \textstyle\begin{cases} \liminf_{t\rightarrow \infty }\frac{1}{t}\mathbb{E}\int _{0}^{t}S( \tau )\,\mathrm{d}\tau \geq \frac{S^{*}}{2}-\limsup_{t\rightarrow \infty }\frac{1}{t}\mathbb{E}\int _{0}^{t} \frac{(S(\tau )-S^{*})^{2}}{2S^{*}}\,\mathrm{d}\tau \geq \frac{S^{*}}{2}- \frac{C_{5}}{2S^{*}C_{6}}, \\ \liminf_{t\rightarrow \infty }\frac{1}{t}\mathbb{E}\int _{0}^{t}I( \tau )\,\mathrm{d}\tau \geq \frac{I^{*}}{2}-\limsup_{t\rightarrow \infty }\frac{1}{t}\mathbb{E}\int _{0}^{t} \frac{(I(\tau )-I^{*})^{2}}{2I^{*}}\,\mathrm{d}\tau \geq \frac{I^{*}}{2}- \frac{C_{5}}{2I^{*}C_{6}}, \\ \liminf_{t\rightarrow \infty }\frac{1}{t}\mathbb{E}\int _{0}^{t}R( \tau )\,\mathrm{d}\tau \geq \frac{R^{*}}{2}-\limsup_{t\rightarrow \infty }\frac{1}{t}\mathbb{E}\int _{0}^{t} \frac{(R(\tau )-R^{*})^{2}}{2R^{*}}\,\mathrm{d}\tau \geq \frac{R^{*}}{2}- \frac{C_{5}}{2R^{*}C_{6}}, \\ \liminf_{t\rightarrow \infty }\frac{1}{t}\mathbb{E}\int _{0}^{t}W( \tau )\,\mathrm{d}\tau \geq \frac{W^{*}}{2}-\limsup_{t\rightarrow \infty }\frac{1}{t}\mathbb{E}\int _{0}^{t} \frac{(W(\tau )-W^{*})^{2}}{2W^{*}}\,\mathrm{d}\tau \geq \frac{W^{*}}{2}- \frac{C_{5}}{2W^{*}C_{6}}. \end{cases} $$

Finally, when $S^{*2}C_{6}>C_{5}$, $I^{*2}C_{6}>C_{5}$, $R^{*2}C_{6}>C_{5}$, $W^{*2}C_{6}>C_{5}$, it yields

22 $$ \textstyle\begin{cases} \liminf_{t\rightarrow \infty }\frac{1}{t}\mathbb{E}\int _{0}^{t}S( \tau )\,\mathrm{d}\tau \geq \frac{S^{*}}{2}-\frac{C_{5}}{2S^{*}C_{6}}>0, \quad \text{a.s.} \\ \liminf_{t\rightarrow \infty }\frac{1}{t}\mathbb{E}\int _{0}^{t}I( \tau )\,\mathrm{d}\tau \geq \frac{I^{*}}{2}-\frac{C_{5}}{2I^{*}C_{6}}>0, \quad \text{a.s.} \\ \liminf_{t\rightarrow \infty }\frac{1}{t}\mathbb{E}\int _{0}^{t}R( \tau )\,\mathrm{d}\tau \geq \frac{R^{*}}{2}-\frac{C_{5}}{2R^{*}C_{6}}>0, \quad \text{a.s.} \\ \liminf_{t\rightarrow \infty }\frac{1}{t}\mathbb{E}\int _{0}^{t}W( \tau )\,\mathrm{d}\tau \geq \frac{W^{*}}{2}-\frac{C_{5}}{2W^{*}C_{6}}>0, \quad \text{a.s.} \end{cases} $$

Based on the above analysis and (22), it can be obtained that system (5) with any given initial value $(S(0), I(0), R(0), W(0), k)\in \mathbb{R}_{+}^{4}\times \mathbb{N}$ is permanent in mean almost surely. □

### Theorem 3.2

*For any initial value* $(S(0), I(0), R(0), W(0), k)\in \mathbb{R}_{+}^{4}\times \mathbb{N}$, *if* $(m_{1}, m_{2})\in \mathcal{D}_{1}\cap \mathcal{D}_{2}\cap \mathcal{D}_{3}\cap \mathcal{D}_{5}$, *where* $\mathcal{D}_{5}$*is defined in* (23), *then*

$$ \limsup_{t\rightarrow \infty }\frac{1}{t}\ln \biggl( \frac{I(t)}{d_{2}+\delta +c}+\frac{C_{8}W(t)}{\rho } \biggr)\leq C_{11}\quad \textit{a.s.}, $$

*where* $C_{11}$*is defined in* (34). *If* $C_{11}<0$, *then* $\lim_{t\rightarrow +\infty }I(t)=0$*almost surely*. *Furthermore*, *the distribution of* $S(t)$*weakly converges to the measure with the density* $\sigma (t)$, *which is defined in* (26).

23 $$ \mathcal{D}_{5}=\biggl\{ (m_{1},m_{2})\Big| \begin{aligned} \max \biggl\{ \frac{qd_{1}}{Q^{2}(\varepsilon )}, \frac{q(2d_{1}-\omega ^{2})}{2Q^{2} (\varepsilon )} \biggr\} < m_{2}< m_{1}\end{aligned} \biggr\} . $$

### Proof

Using Lemma 2.1, Lemma 2.2 and the first equation of system (5), an auxiliary stochastic equation is considered as follows:

24 $$ \mathrm{d}\chi (t)= \biggl(\varLambda (k)-d_{1}\chi (t)+ \frac{m_{2}Q^{2}(\varepsilon )}{q}\chi (t) \biggr)+\omega \chi (t) \,\mathrm{d}B(t)+ \int _{\mathbb{M}}\lambda (u)\chi (t-)\widetilde{U}( \mathrm{d}t, \mathrm{d}u),$$

with the initial condition $\chi (0)=S(0)>0$.

In order to facilitate the following proof, $\nu _{j}(\tau )$ ($j=1,2,3$) on $(0,\infty )$ are defined as follows:

$$\begin{aligned}& \nu _{1}(\tau )=\varLambda (k)-d_{1}\tau + \frac{m_{2}Q^{2}(\varepsilon )}{q}\tau , \qquad \nu _{2}(\tau )=\omega \tau + \int _{\mathbb{M}}\lambda (u)\widetilde{U}(\mathrm{d}\tau , \mathrm{d}u), \\& \nu _{3}=\omega ^{2}+ \biggl( \int _{\mathbb{M}}\bigl[\lambda (\tau )-\ln \bigl(1+ \lambda (\tau ) \bigr)\bigr]\psi \,\mathrm{d}\tau \biggr)^{2}. \end{aligned}$$

Based on simple computations, it can be obtained that

25 $$ \int _{0}^{\infty }\frac{1}{\nu ^{2}_{2}(\tau )}e^{\int _{1}^{\tau } \frac{2\nu _{1}(u)}{\nu ^{2}_{2}(u)}\,\mathrm{d}u} \,\mathrm{d}\tau = \frac{e^{\frac{2\varLambda (k)}{\nu _{3}}}}{\nu _{3}} \int _{0}^{\infty } \tau ^{ (\frac{2qd_{1}-2m_{2}Q^{2}(\varepsilon )}{q\nu _{3}}-2 )}{e^{-\frac{2\varLambda (k)}{\nu _{3}\tau }}} \,\mathrm{d}\tau < \infty . $$

Consequently, it follows from (25) that sufficient conditions in Theorem 1.16 of [46] are satisfied, and it can be obtained that Eq. (24) has a stationary ergodic solution and the invariant density $\sigma (\tau )$ defined on $\tau \in (0, \infty )$ is

26 $$ \sigma (\tau )=C_{7}\nu _{3}^{-1} \tau ^{ ( \frac{2qd_{1}-2m_{2}Q^{2}(\varepsilon )}{q\nu _{3}}-2 )}{e^{- \frac{2\varLambda (k)}{\nu _{3}\tau }}},$$

where $C_{7}= [\nu _{3}^{-1} (\frac{\nu _{3}}{2\varLambda (k)} )^{\frac{2(qd_{1}-m_{2}Q^{2}(\varepsilon ))}{q\nu _{3}}+1} \varGamma (\frac{2(qd_{1}-m_{2}Q^{2}(\varepsilon ))}{q\nu _{3}}+1 ) ]^{-1}$ represents a constant such that $\int _{0}^{\infty }\sigma (\tau )\,\mathrm{d}\tau =1$.

Using the 1-dimensional stochastic differential equation comparison theorem [44], it can be concluded that $S(t)\leq \chi (t)$ holds for any $t\geq 0$ almost surely. Further computations show that

27 $$\begin{aligned}& \begin{aligned}[b] L_{1}&= \int _{0}^{\infty }\tau \sigma (\tau )\,\mathrm{d}\tau = \frac{C_{7}}{\nu _{3}} \int _{0}^{\infty }\tau ^{- \frac{2(qd_{1}-m_{2}Q^{2}(\varepsilon ))}{q\nu _{3}}-1}e^{- \frac{2\varLambda (k)}{\nu _{3}\tau }} \,\mathrm{d}\tau \\ &=\frac{C_{7}}{\nu _{3}} \int _{0}^{\infty } \biggl( \frac{2\varLambda (k)}{\nu _{3}} \biggr)^{- \frac{2(qd_{1}-m_{2}Q^{2}(\varepsilon ))}{q\nu _{3}}-1}\tau ^{ \frac{2(qd_{1}-m_{2}Q^{2}(\varepsilon ))}{q\nu _{3}}-1}e^{-\tau } \biggl( \frac{2\varLambda (k)}{\nu _{3}} \biggr)\,\mathrm{d}\tau \\ &=\frac{C_{7}}{\nu _{3}} \biggl(\frac{\nu _{3}}{2\varLambda (k)} \biggr)^{ \frac{2(qd_{1}-m_{2}Q^{2}(\varepsilon ))}{q\nu _{3}}} \varGamma \biggl( \frac{2(qd_{1}-m_{2}Q^{2}(\varepsilon ))}{q\nu _{3}} \biggr) \\ &=\frac{2\varLambda (k)}{\nu _{3}} \frac{\varGamma (\frac{2(qd_{1}-m_{2}Q^{2}(\varepsilon ))}{q\nu _{3}} )}{\varGamma (\frac{2(qd_{1}-m_{2}Q^{2}(\varepsilon ))}{q\nu _{3}}+1 )} = \frac{q\varLambda (k)}{qd_{1}-m_{2}Q^{2}(\varepsilon )}, \end{aligned} \end{aligned}$$

28 $$\begin{aligned}& \begin{aligned}[b] L_{2}&= \int _{0}^{\infty }\tau ^{2}\sigma (\tau ) \,\mathrm{d}\tau = \frac{C_{7}}{\nu _{3}} \int _{0}^{\infty }\tau ^{- \frac{2(qd_{1}-m_{2}Q^{2}(\varepsilon ))}{q\nu _{3}}}e^{- \frac{2\varLambda (k)}{\nu _{3}\tau }} \,\mathrm{d}\tau \\ &=\frac{C_{7}}{\nu _{3}} \int _{0}^{\infty } \biggl( \frac{2\varLambda (k)}{\nu _{3}} \biggr)^{- \frac{2(qd_{1}-m_{2}Q^{2}(\varepsilon ))}{q\nu _{3}}}\tau ^{ \frac{2(qd_{1}-m_{2}Q^{2}(\varepsilon ))}{q\nu _{3}}-2}e^{-\tau } \biggl( \frac{2\varLambda (k)}{\nu _{3}} \biggr)\,\mathrm{d}\tau \\ &=\frac{C_{7}}{\nu _{3}} \biggl(\frac{\nu _{3}}{2\varLambda (k)} \biggr)^{ \frac{2(qd_{1}-m_{2}Q^{2}(\varepsilon ))}{q\nu _{3}}-1} \varGamma \biggl( \frac{2(qd_{1}-m_{2}Q^{2}(\varepsilon ))}{q\nu _{3}}-1 \biggr) \\ &= \biggl(\frac{2\varLambda (k)}{\nu _{3}} \biggr)^{2} \frac{\varGamma (\frac{2(qd_{1}-m_{2}Q^{2}(\varepsilon ))}{q\nu _{3}}-1 )}{\varGamma (\frac{2(qd_{1}-m_{2}Q^{2}(\varepsilon ))}{q\nu _{3}}+1 )} \\ &=\frac{2q^{2}\varLambda ^{2}(k)}{(qd_{1}-m_{2}Q^{2}(\varepsilon )) [2(qd_{1}-m_{2}Q^{2}(\varepsilon ))-q^{2}\nu _{3}]}. \end{aligned} \end{aligned}$$

Consequently, it follows from (27) and (28) that

29 $$\begin{aligned}& \int _{0}^{\infty } \biggl(\tau - \frac{q\varLambda (k)}{qd_{1}-m_{2}Q^{2}(\varepsilon )} \biggr)^{2} \sigma (\tau )\,\mathrm{d}\tau \\& \quad = \int _{0}^{\infty } \biggl[\tau ^{2}- \frac{2q\varLambda (k)}{qd_{1}-m_{2}Q^{2}(\varepsilon )}\tau + \biggl( \frac{q\varLambda (k)}{qd_{1}-m_{2}Q^{2}(\varepsilon )} \biggr)^{2} \biggr]\sigma (\tau )\,\mathrm{d}\tau \\& \quad = L_{2}-\frac{2q\varLambda (k)}{qd_{1}-m_{2}Q^{2}(\varepsilon )}L_{1}+ \biggl( \frac{q\varLambda (k)}{qd_{1}-m_{2}Q^{2}(\varepsilon )} \biggr)^{2} \\& \quad = \frac{q^{2}\varLambda ^{2}(k)\nu _{3}}{(qd_{1}-m_{2}Q^{2}(\varepsilon ))^{2} [2(qd_{1}-m_{2}Q^{2}(\varepsilon ))-q^{2}\nu _{3}]}. \end{aligned}$$

Define

30 $$ U_{6}(t)=\frac{1}{d_{2}+\delta +c}I(t)+ \frac{1}{\rho }\sqrt{ \frac{\varLambda (k)[m_{1}q+(m_{1}-m_{2})I^{*}]}{d_{1}(d_{2}+\delta +c)(q+I^{*})}}W(t).$$

By using Itô’s formula and Lemma 2.1, we find that

31 $$\begin{aligned} \mathcal{L}(\ln U_{6}) \leq &\frac{[m_{1}(q+Q(\varepsilon ))-m_{2}P(\varepsilon )]W(t)}{(d+\delta +c)(q+Q(\varepsilon ))U_{6}(t)} \biggl[\chi (t)-\frac{q\varLambda (k)}{qd_{1}-m_{2}Q^{2}(\varepsilon )} \biggr] \\ &{}+ \frac{[m_{1}(q+Q(\varepsilon ))-m_{2}P(\varepsilon )]\varLambda (k)W(t)}{d_{1}(q+Q(\varepsilon ))(d_{2}+\delta +c)U_{6}(t)}- \frac{I(t)}{U_{6}(t)} \\ &{}+ \frac{\sqrt{\varLambda (k)[qm_{1}+(m_{1}-m_{2})I^{*}]}}{\sqrt{d_{1}(q+I^{*})(d_{2}+\delta +c)}U_{6}(t)}\bigl[I(t)-W(t)\bigr] \\ \leq &\frac{m_{1}\rho \sqrt{d_{1}(q+I^{*})}}{\sqrt{\varLambda (k)(d_{2}+\delta +c)[m_{1}q+(m_{1}-m_{2})I^{*}]}} \biggl\vert \chi (t)-\frac{q\varLambda (k)}{qd_{1}-m_{2}Q^{2}(\varepsilon )} \biggr\vert \\ &{}- \frac{m_{2}\rho P(\varepsilon )\sqrt{d_{1}(q+I^{*})}}{(q+Q(\varepsilon ))\sqrt{\varLambda (k)(d_{2}+\delta +c)[m_{1}q+(m_{1}-m_{2})I^{*}]}} \biggl\vert \chi (t)-\frac{q\varLambda (k)}{qd_{1}-m_{2}Q^{2}(\varepsilon )} \biggr\vert \\ &{}+\frac{C_{8}-1}{U_{6}(t)} \bigl[I(t)+C_{8}W(t) \bigr] \\ \leq &\frac{m_{1}\rho \sqrt{d_{1}(q+I^{*})}}{\sqrt{\varLambda (k)(d_{2}+\delta +c)[m_{1}q+(m_{1}-m_{2})I^{*}]}} \biggl\vert \chi (t)-\frac{q\varLambda (k)}{qd_{1}-m_{2}Q^{2}(\varepsilon )} \biggr\vert \\ &{}- \frac{m_{2}\rho P(\varepsilon )\sqrt{d_{1}(q+I^{*})}}{(q+Q(\varepsilon ))\sqrt{\varLambda (k)(d_{2}+\delta +c)[m_{1}q+(m_{1}-m_{2})I^{*}]}} \biggl\vert \chi (t)-\frac{q\varLambda (k)}{qd_{1}-m_{2}Q^{2}(\varepsilon )} \biggr\vert \\ &{}+\min \{d_{2}+\delta +c,\rho \}(C_{8}-1)I_{\{C_{8}\leq 1\}}+ \max \{d_{2}+ \delta +c,\rho \}(C_{8}-1)I_{\{C_{8}>1\}}, \end{aligned}$$

where $C_{8}= \frac{\varLambda (k)[m_{1}(q+Q(\varepsilon ))-m_{2}P(\varepsilon )]}{d_{1}(d_{2}+\delta +c)(q+Q(\varepsilon ))}$.

Based on (29) and (31), and integrating (30) from 0 to *t*, we find that

32 $$\begin{aligned}& \frac{\ln U_{6}(t)}{t} \\& \quad \leq \frac{\ln U_{6}(0)}{t}+ \frac{m_{1}\rho \sqrt{d_{1}(q+I^{*})}}{\sqrt{\varLambda (k)(d_{2}+\delta +c)[m_{1}q+(m_{1}-m_{2})I^{*}]}t} \\& \qquad {}\times\int _{0}^{t} \biggl\vert \chi (\tau )- \frac{q\varLambda (k)}{qd_{1}-m_{2}Q^{2}(\varepsilon )} \biggr\vert \,\mathrm{d}\tau \\& \qquad {}- \frac{m_{2}\rho P(\varepsilon )\sqrt{d_{1}(q+I^{*})}}{(q+Q(\varepsilon )) \sqrt{\varLambda (k)(d_{2}+\delta +c)[m_{1}q+(m_{1}-m_{2})I^{*}]}t} \\& \qquad {}\times\int _{0}^{t} \biggl\vert \chi (\tau )- \frac{q\varLambda (k)}{qd_{1}-m_{2}Q^{2}(\varepsilon )} \biggr\vert \,\mathrm{d}\tau \\& \qquad {}+\min \{d_{2}+\delta +c,\rho \}(C_{8}-1)I_{\{C_{8}\leq 1\}}+ \max \{d_{2}+ \delta +c,\rho \}(C_{8}-1)I_{\{C_{8}>1\}}. \end{aligned}$$

According to the ergodicity of $\chi (t)$ and $\int _{0}^{\infty }\tau \sigma (\tau )\,\mathrm{d}\tau <\infty $, it yields that

33 $$\begin{aligned}& \lim_{t\rightarrow \infty } \frac{1}{t} \int _{0}^{t} \biggl\vert \chi ( \tau )- \frac{q\varLambda (k)}{qd_{1}-m_{2}Q^{2}(\varepsilon )} \biggr\vert \,\mathrm{d}\tau \\& \quad = \int _{0}^{\infty } \biggl\vert \tau - \frac{q\varLambda (k)}{qd_{1}-m_{2}Q^{2}(\varepsilon )} \biggr\vert \sigma ( \tau )\,\mathrm{d}\tau \leq \sqrt{ \int _{0}^{\infty } \biggl[\tau - \frac{q\varLambda (k)}{qd_{1}-m_{2}Q^{2}(\varepsilon )} \biggr]^{2} \sigma (\tau )\,\mathrm{d}\tau }. \end{aligned}$$

Based on (29), (31), (32) and (33), it can be obtained that

34 $$\begin{aligned} \limsup_{t\rightarrow \infty } \frac{\ln U_{6}(t)}{t} \leq &\min \{d_{2}+ \delta +c,\rho \}(C_{8}-1)I_{\{C_{8}\leq 1\}}+\max \{d_{2}+\delta +c, \rho \}(C_{8}-1)I_{\{C_{8}>1\}} \\ &{}+ \frac{m_{1}\rho \sqrt{d_{1}(q+I^{*})}}{\sqrt{\varLambda (k)(d_{2}+\delta +c)[m_{1}q+(m_{1}-m_{2})I^{*}]}}C_{9} \\ &{}- \frac{m_{2}\rho P(\varepsilon )\sqrt{d_{1}(q+I^{*})}}{(q+Q(\varepsilon ))\sqrt{\varLambda (k)(d_{2}+\delta +c)[m_{1}q+(m_{1}-m_{2})I^{*}]}}C_{9} \\ =&\min \{d_{2}+\delta +c,\rho \}(C_{8}-1)I_{\{C_{8}\leq 1\}} \\ &{}+\max \{d_{2}+\delta +c,\rho \}(C_{8}-1)I_{\{C_{8}>1\}}+C_{10} \\ :=&C_{11}, \end{aligned}$$

where $C_{9}=\sqrt{ \frac{q^{3}\omega ^{2}\varLambda ^{2}(k)}{(qd_{1}-m_{2}Q^{2}(\varepsilon ))^{2}[q(2d_{1}-\omega ^{2})-2m_{2}Q^{2}(\varepsilon )]}}$, and $C_{10}= \frac{\rho (qd_{1}-m_{2}Q^{2}(\varepsilon ))C_{8}C_{9}}{q\varLambda (k)}$.

If $C_{11}<0$ (defined in (34)) and $(m_{1}, m_{2})\in \mathcal{D}_{1}\cap \mathcal{D}_{2}\cap \mathcal{D}_{3}\cap \mathcal{D}_{5}$, where $\mathcal{D}_{5}$ is defined in (23), then it can be concluded that $\limsup_{t\rightarrow \infty }\frac{\ln I(t)}{t}<0$ almost surely, which reveals that $\lim_{t\rightarrow \infty }I(t)=0$ almost surely. Hence, it completes the proof. □

### Theorem 3.3

*For any initial value* $(S(0), I(0), R(0), W(0), k)\in \mathbb{R}_{+}^{4}\times \mathbb{N}$, *if* $(m_{1}, m_{2})\in \mathcal{D}_{1}\cap \mathcal{D}_{6}$, *where*

35 $$ \mathcal{D}_{6}=\left \{ (m_{1},m_{2})\left | \begin{aligned} &\max \biggl\{ \frac{qd_{1}}{Q^{2}(\varepsilon )}, \frac{q(2d_{1}-\omega ^{2})}{2Q^{2}(\varepsilon )} \biggr\} < m_{2}< m_{1}, \\ &m_{1}> \frac{2\varLambda (k)+\omega ^{2}P(\varepsilon )+2(m_{2}Q(\varepsilon )-d_{1}-\upsilon )P(\varepsilon )}{2P^{2}(\varepsilon )}\end{aligned} \right . \right \} , $$

*then* $\lim_{t\rightarrow +\infty }S(t)=0$*and* $\lim_{t\rightarrow +\infty }I(t)=0$*almost surely*.

### Proof

Firstly, by applying Itô’s formula into the first equation of system (5), we obtain that

36 $$\begin{aligned} \mathrm{d}\ln S(t) =& \biggl[\frac{\varLambda (k)}{S(t)}-d_{1}- \biggl(m_{1}- \frac{m_{2}I(t)}{q+I(t)} \biggr)W(t) \biggr]\,\mathrm{d}t \\ &{}+ \biggl[\frac{\omega }{2}+ \int _{\mathbb{M}}\bigl[\lambda (u)-\ln \bigl(1+ \lambda (u)\bigr) \bigr]\psi \,\mathrm{d}u \biggr]\,\mathrm{d}t \\ &{}+ \omega \,\mathrm{d}B(t)+ \int _{\mathbb{M}}\ln \bigl(1+\lambda (u)\bigr) \widetilde{U}( \mathrm{d}t,\mathrm{d}u). \end{aligned}$$

By integrating both sides of (36) from 0 to *t*, it follows from Lemma 2.1 that

37 $$\begin{aligned} \ln S(t)-\ln S(0) \leq & \biggl( \frac{\varLambda (k)}{P(\varepsilon )}-d_{1}-m_{1}P( \varepsilon )+m_{2}Q(\varepsilon )+\frac{\omega ^{2}}{2}+\upsilon \biggr)t \\ &{}+\omega B(t)+ \int _{0}^{t} \int _{\mathbb{M}}\ln \bigl(1+\lambda (u)\bigr) \widetilde{U}( \mathrm{d}\tau ,\mathrm{d}u)\,\mathrm{d}\tau . \end{aligned}$$

Let $F(t)=\int _{0}^{t}\int _{\mathbb{M}}\ln (1+\lambda (u))\widetilde{U}( \mathrm{d}\tau ,\mathrm{d}u)\,\mathrm{d}\tau $. It can be shown that

$$ \bigl\langle F(t), F(t)\bigr\rangle = \int _{0}^{t} \biggl[ \int _{\mathbb{M}}\ln \bigl(1+ \lambda (u)\bigr)\widetilde{U}( \mathrm{d}\tau ,\mathrm{d}u) \biggr]^{2} \,\mathrm{d}\tau $$

and that

$$ \mathbb{P} \biggl\{ \sup_{0\leq t\leq T_{k}} \biggl[F(t)- \frac{1}{2} \bigl\langle F(t), F(t)\bigr\rangle \biggr]>2\ln T_{k} \biggr\} \leq \frac{1}{T_{k}^{2}} $$

based on the exponential martingales inequality.

According to the Borel–Cantelli lemma [45], it can be concluded that a random integer $T_{k0}=T_{k0}(\omega )$ exists for almost all $\omega \in \varOmega $, yielding that

38 $$ \sup_{0\leq t\leq T_{k}} \biggl[F(t)- \frac{1}{2}\bigl\langle F(t), F(t) \bigr\rangle \biggr]\leq 2\ln T_{k}$$

holds for $T_{k}\geq T_{k0}$ almost surely. It follows from (38) that

39 $$ F(t)\leq 2\ln T_{k}+\frac{1}{2}\bigl\langle F(t), F(t)\bigr\rangle $$

holds for all $0\leq t\leq T_{k}$ almost surely.

Substituting (39) into (37), it can be obtained that

$$\begin{aligned} \ln S(t)-\ln S(0) \leq & \biggl( \frac{\varLambda (k)}{P(\varepsilon )}-d_{1}-m_{1}P( \varepsilon ) +m_{2}Q(\varepsilon )+\frac{\omega ^{2}}{2}+\upsilon \biggr)t \\ &{}+\omega B(t)+2\ln T_{k} \end{aligned}$$

holds for all $0\leq t\leq T_{k}$ almost surely. Furthermore, it can be shown that

40 $$\begin{aligned} \frac{\ln S(t)-\ln S(0)}{t} \leq & \frac{\varLambda (k)}{P(\varepsilon )}-d_{1}-m_{1}P(\varepsilon )+m_{2}Q( \varepsilon ) \\ &{}+\frac{\omega ^{2}}{2}+\upsilon +\frac{\omega B(t)}{t}+ \frac{2\ln T_{k}}{T_{k}-1} \end{aligned}$$

holds for $0\leq T_{k}-1\leq t\leq T_{k}$ almost surely.

It is easy to show that $\lim_{t\rightarrow \infty }\frac{B(t)}{t}=0$ almost surely. If $(m_{1},m_{2})\in \mathcal{D}_{1}\cap \mathcal{D}_{6}$, then, following (40),

$$ \limsup_{t\rightarrow \infty }\frac{\ln S(t)}{t}\leq \frac{\varLambda (k)}{P(\varepsilon )}-d_{1}-m_{1}P( \varepsilon )+m_{2}Q( \varepsilon )+\frac{\omega ^{2}}{2}+\upsilon < 0 $$

yields that

41 $$ \lim_{t\rightarrow \infty }S(t)=0\quad \text{a.s.} $$

Secondly, using similar proofs to Theorem 3.2 of this paper, if $(m_{1},m_{2})\in \mathcal{D}_{1}\cap \mathcal{D}_{6}$, then it can be shown that

42 $$ \lim_{t\rightarrow \infty }I(t)=0\quad \text{a.s.} $$

Hence, it completes the proof. □

### Remark 3.4

Following similar arguments given in [17, 40], we can show that the basic reproduction numbers for the deterministic and stochastic versions of system (5) are obtained as follows:

$$ \mathcal{R}_{0}^{d}=\frac{m_{1}\varLambda (k)}{d_{1}(d_{2}+\delta +c)} \quad \text{and}\quad \mathcal{R}_{0}^{s}= \frac{d_{1} \mathcal{R}_{0}^{d}}{ (d_{1}+\frac{\omega ^{2}}{2}+ \int _{\mathbb{M}}[\lambda (u)-\ln (1+\lambda (u))]\psi \,\mathrm{d}u )}, $$

respectively. Note that $\mathcal{R}_{0}^{s}<\mathcal{R}_{0}^{d}$ and that $\mathcal{R}_{0}^{s}$ decreases when the intensity of the Lévy jump increases.

### Remark 3.5

Based on the mathematical formulation of system (5), it can be concluded that the state variable $R(t)$ does not impose dynamic effects on infectious disease transmission. Hence, we have discussed some sufficient conditions for disease extinction omitting $R(t)$ in Theorems 3.2 and 3.3 of this paper.

## Numerical simulation

Simulation studies are used to explore the combined dynamic effects of Markovian switching and media coverage on the stochastic epidemiological dynamics of system (5). Calculations are based on Milstein’s higher order method [46]. Suppose state space $\mathbb{N}=\{1,2\}$. Using the Markovian chain law, system (5) can be investigated as a hybrid system switching between subsystems

43 $$ \textstyle\begin{cases} \mathrm{d}{S}(t)= [\varLambda (1)-d_{1} S(t)- (m_{1}- \frac{m_{2}I(t)}{q+I(t)} )S(t)W(t) ]\,\mathrm{d}t \\ \hphantom{\mathrm{d}{S}(t)={}}{} +\omega S(t)\,\mathrm{d}B(t)+\int _{\mathbb{M}} \lambda (u)S(t-)\widetilde{U}(\mathrm{d}t,\mathrm{d}u), \\ \mathrm{d}{I}(t)= [ (m_{1}-\frac{m_{2}I(t)}{q+I(t)} )S(t)W(t)-(d_{2}+ \delta +c)I(t) ]\,\mathrm{d}t, \\ \mathrm{d}{R}(t)= [\delta I(t)-d_{3} R(t) ]\,\mathrm{d}t, \\ \mathrm{d}W(t)=\rho [I(t)-W(t) ]\,\mathrm{d}t, \end{cases} $$

and

44 $$ \textstyle\begin{cases} \mathrm{d}{S}(t)= [\varLambda (2)-d_{1} S(t)- (m_{1}- \frac{m_{2}I(t)}{q+I(t)} )S(t)W(t) ]\,\mathrm{d}t \\ \hphantom{\mathrm{d}{S}(t)={}}{} +\omega S(t)\,\mathrm{d}B(t)+\int _{\mathbb{M}} \lambda (u)S(t-)\widetilde{U}(\mathrm{d}t,\mathrm{d}u), \\ \mathrm{d}{I}(t)= [ (m_{1}-\frac{m_{2}I(t)}{q+I(t)} )S(t)W(t)-(d_{2}+ \delta +c)I(t) ]\,\mathrm{d}t, \\ \mathrm{d}{R}(t)= [\delta I(t)-d_{3} R(t) ]\,\mathrm{d}t, \\ \mathrm{d}W(t)=\rho [I(t)-W(t) ]\,\mathrm{d}t. \end{cases} $$

In the following numerical examples we take $d_{1}=0.4$, $q=0.1$, $\omega ^{2}=0.25$, $d_{2}=0.2$, $\delta =0.3$, $c=0.1$, $d_{3}=0.05$, $\rho =0.5$ with appropriate units. Parameters *λ*, $m_{1}$ and $m_{2}$ are varied.

**Figure 1:**
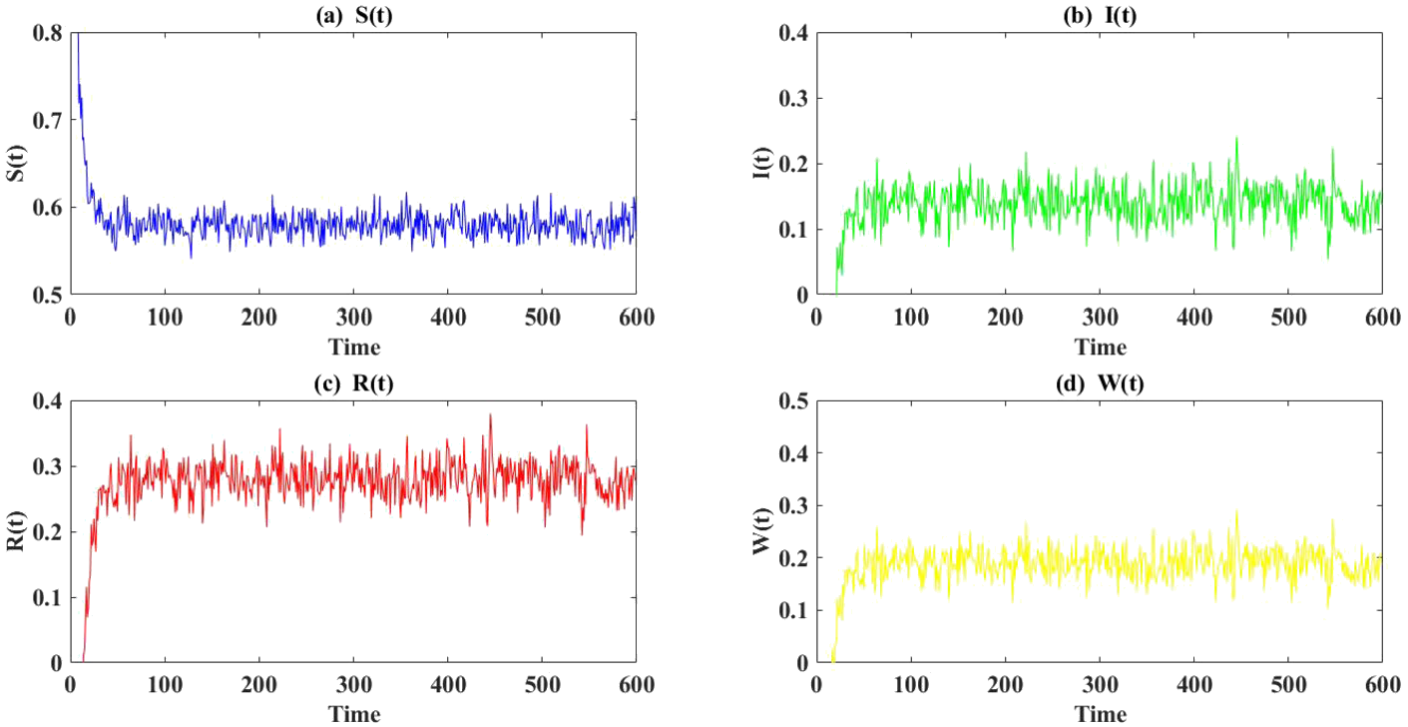
Existence of the endemic equilibrium and permanence in mean almost surely. Parameter values: $\lambda =0.01$, $m_{1}=0.3$ and $m_{2}=0.15$

In Fig. 1 we give an example of persistence in mean. Here, $\lambda =0.01$, $m_{1}=0.3$ and $m_{2}=0.15$. It follows from (6) and simple computations that the endemic equilibrium of the deterministic version of system (5) exists. These parameter values also satisfy the existence of a stationary distribution when $(m_{1},m_{2})\in \mathcal{D}_{1}\cap \mathcal{D}_{2}\cap \mathcal{D}_{3}= \{(m_{1}, m_{2})|0.0747< m_{2}< m_{1}<0.5333\}$. Here, $(S^{*}, I^{*}, R^{*}, W^{*})=(0.5761, 0.1428, 0.2811, 0.2811)$, $(m_{1}, m_{2})=(0.3,0.15)\in \mathcal{D}_{1}\cap \mathcal{D}_{2} \cap \mathcal{D}_{3}\cap \mathcal{D}_{4}$ is satisfied, and sufficient conditions in Theorem 3.1 hold. Additionally, we have $\limsup_{t\rightarrow \infty }\frac{1}{t}\mathbb{E}\int _{0}^{t}[(S( \tau )-S^{*})^{2}+(I(\tau )-I^{*})^{2}+(R(\tau )-R^{*})^{2}+(W(\tau )-W^{*})^{2}] \,\mathrm{d}\tau \leq 0.9473$, and it can be concluded that system (5) is permanent in mean (based on Theorem 3.1).

**Figure 2:**
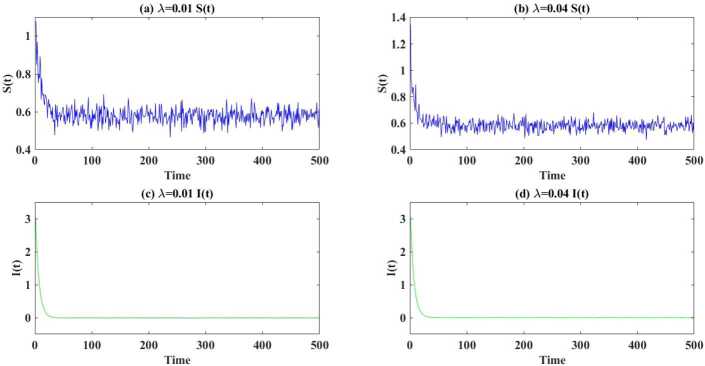
Examples of Theorem 3.2. The dynamics of system (43) are shown with parameter values as described in the text, and (left column) $\lambda =0.01$, $m_{1}=0.4$ and $m_{2}=0.25$ (right column) $\lambda =0.04$, $m_{1}=0.5$ and $m_{2}=0.2$

Figure 2 provides two examples satisfying Theorem 3.2 given system (43). Here we assume (left column) $\lambda =0.01$, $m_{1}=0.4$ and $m_{2}=0.25$, and (right column) $\lambda =0.04$, $m_{1}=0.5$ and $m_{2}=0.2$. Given $\lambda =0.01$, we see that $(m_{1},m_{2})\in \mathcal{D}_{1}\cap \mathcal{D}_{2}\cap \mathcal{D}_{3} \cap \mathcal{D}_{5}=\{(m_{1},m_{2})|0.1926< m_{2}< m_{1}<0.5333\}$, and the distribution of $S(t)$ weakly converges to the measure with $\sigma (t)$ defined in (23) and $\lim_{t\rightarrow \infty } I(t)=0$ almost surely. When $\lambda =0.04$, $(m_{1},m_{2})\in \mathcal{D}_{1}\cap \mathcal{D}_{2}\cap \mathcal{D}_{3} \cap \mathcal{D}_{5}=\{(m_{1},m_{2})|0.1631< m_{2}< m_{1}<0.5333\}$ is satisfied, and we obtain the same result.

**Figure 3:**
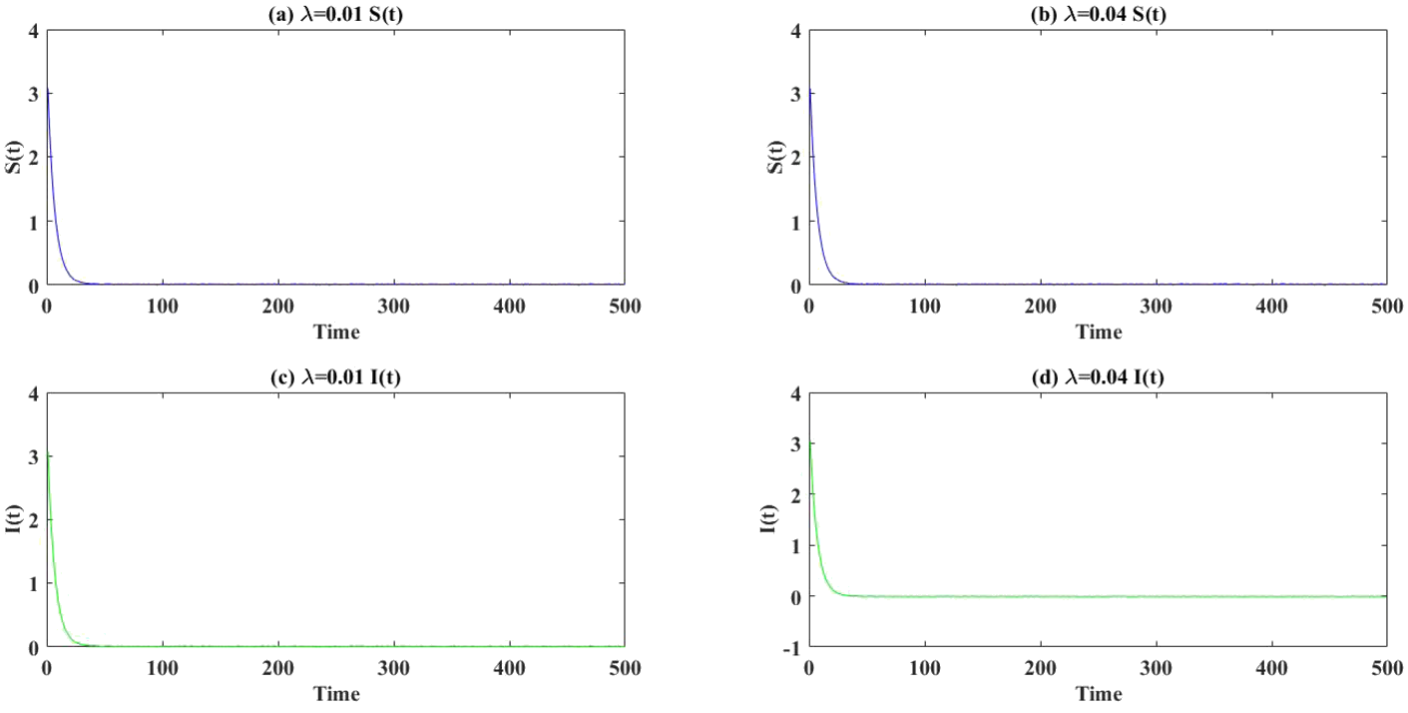
Examples of Theorem 3.3. The dynamics of system (44) are shown with parameter values as described in the text, and (left column) $\lambda =0.01$, $m_{1}=0.8$ and $m_{2}=0.25$ (right column) $\lambda =0.04$, $m_{1}=0.9$ and $m_{2}=0.2$

Two examples of Theorem 3.3 are shown in Fig. 3 given system (44). When $\lambda =0.01$, extinction of all individuals requires that $(m_{1}, m_{2})\in \mathcal{D}_{1}\cap \mathcal{D}_{6}=\{(m_{1},m_{2}) |0.1926< m_{2}< m_{1},m_{1}>0.7442 \}$. This is shown to be true in the left column of Fig. 3 with $m_{1}=0.8$ and $m_{2}=0.25$). In the right column we see that extinction is accomplished with probability 1 when $\lambda =0.04$, $m_{1}=0.9$ and $m_{2}=0.2$, since $(m_{1},m_{2})\in (m_{1}, m_{2})\in \mathcal{D}_{1}\cap \mathcal{D}_{6}= \{(m_{1},m_{2})|0.1926< m_{2}< m_{1},m_{1}>0.8721\}$ is satisfied.

## Conclusion

It is well known that the studies of stochastic perturbations and media coverage are two important and well-established disciplines in mathematical epidemiology [1, 3, 47, 48]. Here, we have extended the model in [17] to include Markovian switching, telephone noise, Lévy jumps and media impact. These extensions have been motivated by the following facts:

(I) Lévy jumps have been shown to effectively portray an unexpected outbreak of infectious disease and other sudden severe perturbations arising in the real world [23–28], which cannot be accurately depicted by Brownian motion;
(II) Evidences from real-world observations point out that the birth rate of susceptible individuals is subject to both white noise and telephone noise [18–22] (which is generally memoryless and can be regarded as a switching state among some considerable environmental regimes [18–20]);
(III) It is well known that there is a profound relationship between public health issues and mass media coverage, and that media reports can elicit changes in individual behaviour during an infectious disease outbreak, affecting the implementation of public health measures to mitigate infection [29, 43].

Based on the theoretical findings in Lemma 2.1 and Lemma 2.2, if intensities of media coverage are restricted within certain ranges, then the solution is stochastically ultimately bounded, and there exists a globally unique positive solution to the proposed system. Furthermore, it shows that there exists a stationary distribution to the positive solution (a stationary Markov process) when intensities of media coverage and Markovian switching are restricted within certain ranges. All these theoretical findings can be found in Theorem 2.3.

Some sufficient conditions associated with Markovian switching, Lévy jumps and media coverage are derived for the persistence in mean of each individual and extinction of the infectious disease, which are discussed in Theorems 3.1, 3.2 and 3.3. Corresponding numerical experiments and corresponding figures indicate that permanence in mean of each individual and extinction of disease have strong relationship with intensities of media coverage and Lévy jumps. Furthermore, the basic reproduction numbers of the deterministic and stochastic version are obtained in Remark 3.4, which reveals that $\mathcal{R}_{0}^{s}<\mathcal{R}_{0}^{d}$ and that $\mathcal{R}_{0}^{s}$ decreases when the intensity of the Lévy jump increases.

Compared with the recent related work, the combined dynamic effects of Markovian switching and media coverage on a stochastic epidemiological system with Lévy jumps and distributed delay are investigated in this paper, which has not been studied before. Our analytical findings thus provide enhanced knowledge in the field of mathematical epidemiology.

## Appendix A: Proof of Lemma 2.1

### Proof

Let $\alpha _{1}(t)=S^{\gamma }(t)$, $0<\gamma <1$. By applying Itô’s formula [45] to $e^{t}\alpha _{1}(t)$, it can be obtained that

45 $$\begin{aligned}& \mathrm{d}\bigl(e^{t}\alpha _{1}(t)\bigr) \\& \quad = e^{t} \biggl[\alpha _{1}(t)+\gamma \alpha _{1}^{\gamma -1} \,\mathrm{d}\alpha _{1}(t)+ \frac{\gamma (\gamma -1)\alpha _{1}^{\gamma -2}(t)(\mathrm{d}\alpha _{1})^{2}}{2} \biggr] \\& \quad = e^{t} \biggl\{ 1+\gamma \biggl[\varLambda (k)-d_{1}S(t)- \biggl(m_{1}- \frac{m_{2}I(t)}{q+I(t)} \biggr)S(t)W(t) \biggr] \biggr\} \alpha _{1}(t) \\& \qquad {}+e^{t} \biggl[\gamma (\gamma -1)\omega ^{2}+ \int _{\mathbb{M}}\bigl[\bigl(1+ \lambda (u)\bigr)^{\gamma }-1- \gamma \lambda (u)\bigr]\psi \,\mathrm{d}u \biggr] \alpha _{1}(t) \\& \qquad {}+e^{t} \biggl[\gamma \omega \,\mathrm{d}B(t)+ \int _{\mathbb{M}}\bigl[\bigl(1+ \lambda (u)\bigr)^{\gamma }-1 \bigr]\widetilde{U}(\mathrm{d}t,\mathrm{d}u) \biggr] \alpha _{1}(t). \end{aligned}$$

By integrating (45) from 0 to *t* and taking expectations on both sides of (45), it yields that

46 $$\begin{aligned}& \mathbb{E}\bigl(e^{t}\alpha _{1}(t)\bigr) \\& \quad = \alpha _{1}(0)+\mathbb{E} \int _{0}^{t}e^{\tau } \biggl\{ 1+ \gamma \biggl[\varLambda (k)-d_{1}S(t)- \biggl(m_{1}- \frac{m_{2}I(t)}{q+I(t)} \biggr)S(t)W(t) \biggr] \biggr\} \alpha _{1}( \tau )\,\mathrm{d}\tau \\& \qquad {}+\mathbb{E} \int _{0}^{t}e^{\tau } \biggl[ \int _{\mathbb{M}}\bigl[\bigl(1+ \lambda (u)\bigr)^{\gamma }-1 \bigr]\widetilde{U}(\mathrm{d}t,\mathrm{d}u)-\gamma (1- \gamma )\omega ^{2} \biggr]\alpha _{1}(\tau )\,\mathrm{d}\tau \\& \quad \leq \alpha _{1}(0)+\mathbb{E} \int _{0}^{t}e^{\tau } \bigl[1+ \varLambda (k)\gamma -d_{1}\gamma S(t)-(m_{1}-m_{2})S(t)W(t)- \gamma (1- \gamma )\omega ^{2} \bigr]\alpha _{1}(\tau )\, \mathrm{d}\tau \\& \qquad {}+\mathbb{E} \int _{0}^{t}e^{\tau } \biggl[ \int _{\mathbb{M}}\bigl[\bigl(1+ \lambda (u)\bigr)^{\gamma }-1- \gamma \lambda (u)\bigr]\psi \,\mathrm{d}u \biggr] \alpha _{1}(\tau ) \,\mathrm{d}\tau . \end{aligned}$$

For $\alpha _{1}\geq 0$ and $0<\gamma <1$, $\alpha _{1}^{\gamma }\leq 1+\gamma (\alpha _{1}-1)$ holds. Consequently, it can be shown that

47 $$\begin{aligned}& \alpha _{1}(t) \biggl[1+\varLambda (k) \gamma -d_{1}\gamma S(t)-\gamma (1- \gamma )\omega ^{2}+ \int _{\mathbb{M}}\bigl[\bigl(1+\lambda (u)\bigr)^{\gamma }-1- \gamma \lambda (u)\bigr]\psi \,\mathrm{d}u \biggr] \\& \quad \leq \bigl[1+\varLambda (k)\gamma -d_{1}\gamma S(t) \bigr]\alpha _{1}(t) \leq Q_{1}(\gamma ), \end{aligned}$$

where $Q_{1}(\gamma )$ represents a positive constant associated with *γ*. Hence, it can be concluded that

48 $$ \mathbb{E}\bigl(e^{t}\alpha _{1}(t) \bigr)\leq \alpha _{1}(0)+\mathbb{E} \int _{0}^{t}e^{ \tau }Q_{1}( \gamma )\,\mathrm{d}\tau . $$

Based on (48), it can be shown that

49 $$ \limsup_{t\rightarrow \infty }\mathbb{E} \bigl(S^{\gamma }(t)\bigr)\leq Q_{1}( \gamma ). $$

When $(m_{1},m_{2})\in \mathcal{D}_{1}$, it follows from the standard arguments that

50 $$ \textstyle\begin{cases} \lim_{t\rightarrow \infty }I(t)\leq \frac{m_{1}Q_{1}(\gamma )-(d_{2}+\delta +c)}{m_{2}Q_{1}(\gamma )}:=Q_{2}( \gamma ), \\ \lim_{t\rightarrow \infty }R(t)\leq \frac{\delta Q_{2}(\gamma )}{d_{3}}:=Q_{3}(\gamma ). \end{cases} $$

Based on the mathematical formulation of $W(t)$, we find that

51 $$ \limsup_{t\rightarrow \infty }W(t)\leq \limsup _{t\rightarrow \infty }I(t) \leq Q_{2}(\gamma ). $$

Let $\tilde{\alpha }(t)=(S(t), I(t), R(t), W(t), k)^{T}\in \mathbb{R}_{+}^{4} \times \mathbb{N}$, then

52 $$ 2^{(1-\frac{\gamma }{2})\wedge 0} \bigl\vert \tilde{\alpha }(t) \bigr\vert ^{\gamma }\leq S^{ \gamma }(t)+I^{\gamma }(t)+R^{\gamma }(t)+W^{\gamma }(t). $$

According to (49), (50), (51), and (52), it can be obtained that

53 $$\begin{aligned} \limsup_{t\rightarrow \infty }\mathbb{E} \bigl\vert \tilde{\alpha }(t) \bigr\vert ^{\gamma } \leq &0.5^{(1-\frac{\gamma }{2})\wedge 0}\limsup _{t\rightarrow \infty } \mathbb{E}\bigl[S^{\gamma }(t)+I^{\gamma }(t)+R^{\gamma }(t)+W^{\gamma }(t) \bigr] \\ \leq &0.5^{(1-\frac{\gamma }{2})\wedge 0}\bigl[Q_{1}(\gamma )+2Q_{2}( \gamma )+Q_{3}(\gamma )\bigr]:=Q(\gamma ). \end{aligned}$$

Assume $Q(\varepsilon )= (\frac{Q(\gamma )}{\varepsilon } )^{ \frac{1}{\gamma }}$, where $0<\varepsilon <1$ denotes an arbitrarily small constant. Based on Chebyshev’s inequality, it can be concluded that

54 $$ \textstyle\begin{cases} \mathbb{P}[\tilde{\alpha }(t)< Q(\varepsilon )]\leq Q(\varepsilon )^{ \gamma }\mathbb{P}[\tilde{\alpha }^{-\gamma }(t)], \\ \liminf_{t\rightarrow \infty }\mathbb{P}[\tilde{\alpha }(t)\leq Q( \varepsilon )]\geq 1-\varepsilon . \end{cases} $$

According to Chebyshev’s inequality and (54), it can be shown that there exists $P(\varepsilon )>0$ such that

55 $$ \liminf_{t\rightarrow \infty }\mathbb{P}\bigl[\tilde{\alpha }(t)\geq P( \varepsilon )\bigr]\geq 1-\varepsilon . $$

Based on (54) and (55), it completes the proof. □

## Appendix B: Proof of Lemma 2.2

### Proof

According to similar arguments utilised in [17, 40], it is straightforward to show the existence of a unique local positive solution $(S(t), I(t), R(t), W(t), k)\in \mathbb{R}_{+}^{4}\times \mathbb{N}$ on $t\in [0, T_{e})$ almost surely for any initial value.

Let $T_{e}$ stand for explosion time [45]. Assume $n_{0}\geq 1$ represents a sufficiently large integer such that $(S(0), I(0), R(0), W(0))$ all lie within $[\frac{1}{n_{0}}, n_{0}]$. For any integer $n\geq n_{0}$, the stopping time [45] can be defined as follows:

$$ T_{s}=\inf \left \{ t\in [0,T_{e})\left | \begin{aligned} &\min \bigl\{ S(t), I(t), R(t), W(t)\bigr\} \leq \frac{1}{n},\text{or} \\ &\max \bigl\{ S(t), I(t), R(t), W(t)\bigr\} \geq n\end{aligned} \right . \right \} . $$

It follows from the mathematical formulation of $T_{s}$ that $T_{s}$ is increasing when $n\rightarrow \infty $. Set $T_{\infty }=\lim_{n\rightarrow \infty }T_{s}$. Then it is easy to show that $T_{\infty }\leq T_{e}$ almost surely. Further computations show that $T_{e}=\infty $ almost surely when $T_{\infty }=\infty $ holds almost surely, which yields $(S(t), I(t), R(t), W(t), k)\in \mathbb{R}_{+}^{4}\times \mathbb{N}$ hold for all $t\geq 0$. Hence, we will show that $T_{\infty }=\infty $ almost surely.

If $T_{\infty }=\infty $ almost surely does not hold, then there exists a pair of positive constants $\tilde{N}_{0}>0$ and $0<\epsilon <1$ such that $\mathbb{P}\{T_{\infty }\leq \tilde{N}_{0}\}\geq \epsilon $. Hence, there exists a positive integer $n_{1}\geq n_{0}$ such that $\mathbb{P}\{T_{s}\leq \tilde{N}_{0}\}\geq \epsilon $ holds for any $n\geq n_{1}$. By defining a $C^{4}$-function $W:\mathbb{R}_{+}^{4}\rightarrow \mathbb{R}_{+}\cup \{0\}$ as follows:

$$ V(S,I,R,W)=S-\ln S+I-\ln I+R-\ln R-3+\frac{1}{\rho } (W-1-\ln W ). $$

Using Itô’s formula [45] and calculating the derivation of $\mathrm{d}V(S,I,R,W)$ along the solution of system (5), we find that

$$\begin{aligned}& \mathrm{d}V(S,I,R,W) \\& \quad = \biggl(1-\frac{1}{S(t)} \biggr) \biggl[\varLambda (k)-d_{1}S(t)- \biggl(m_{1}- \frac{m_{2}I(t)}{q+I(t)} \biggr)S(t)W(t) \biggr]\, \mathrm{d}t \\& \qquad {}+ \biggl(1-\frac{1}{I(t)} \biggr) \biggl[ \biggl(m_{1}- \frac{m_{2}I(t)}{q+I(t)} \biggr)S(t)W(t)-(d_{2}+\delta +c)I(t) \biggr] \, \mathrm{d}t \\& \qquad {}+ \biggl(1-\frac{1}{R(t)} \biggr) \bigl[\delta I(t)-d_{3}R(t) \bigr] \,\mathrm{d}t+\rho \biggl(1-\frac{1}{W(t)} \biggr) \bigl[I(t)-W(t) \bigr]\,\mathrm{d}t \\& \qquad {}+\bigl(S(t)-1\bigr)\omega \,\mathrm{d}B(t)+ \biggl[\frac{\omega ^{2}}{2}+ \int _{ \mathbb{M}}(\lambda (u)-\ln \bigl(1+\lambda (u)\bigr)\psi \, \mathrm{d}u \biggr] \,\mathrm{d}t \\& \qquad {}+ \int _{\mathbb{M}}\bigl[\lambda (u)S(t-)-\ln \bigl(1+\lambda (u) \bigr)\bigr] \widetilde{U}(\mathrm{d}t,\mathrm{d}u) \\& \quad =\mathcal{L}V\,\mathrm{d}t+\bigl(S(t)-1\bigr)\omega \,\mathrm{d}B(t)+ \int _{ \mathbb{M}}\bigl[\lambda (u)S(t-)-\ln \bigl(1+\lambda (u) \bigr)\bigr]\widetilde{U}( \mathrm{d}t,\mathrm{d}u). \end{aligned}$$

When hypotheses (H2) and (H3) hold, it yields from Lemma 2.1 that

56 $$\begin{aligned} \mathcal{L}V \leq &\varLambda (k)+d_{1}+d_{2}+d_{3}+\delta +c+\rho +(m_{1}+ \rho +\delta )Q(\varepsilon ) \\ &{}+\frac{(1+m_{1}q)Q^{2}(\varepsilon )}{q}+ \frac{m_{2}Q^{3}(\varepsilon )}{q}+\frac{\omega ^{2}}{2}+\upsilon . \end{aligned}$$

The following arguments are similar to those in [17, 40], which are omitted here. Based on the above analysis, it can be concluded that $\tau _{\infty }=\infty $. □
